# Bone marrow derived mesenchymal stem cells improve acute lung injury induced by sepsis in rats

**DOI:** 10.1186/2197-425X-3-S1-A638

**Published:** 2015-10-01

**Authors:** S Huang, M Yao

**Affiliations:** The First Affiliated Hospital of Sun Yat-sen University, Guangzhou, China

## Introduction

Acute Respiratory Distress Syndrome(ARDS) is a common respiratory critical illness, usually develops in patients with predisposing conditions that induce systemic inflammatory response, such as sepsis. This study was undertaken to examine the effects of BMSCs injection on ARDS induced by sepsis.

## Objectives

To assess the effect of mesenchymal stem cells (MSCs) on acute lung injury induced by sepsis.

## Methods

There were 25 rats in each group, including sepsis group, sepsis+antibiotic group, sepsis+antibiotic+MSCs group and sham group, twenty of which being sacrificed at 12 h, 18 h, 24 h and 48 h after surgery, respectively, five for survival analysis, and 5 normal controls. Survival rate, pulmonary physiological function, alveolar capillary barrier, lung inflammatory reaction and pathological injury were measured.

## Results

①at 18 h, total nucleated cell count of bronchoalveolar lavage fluid was 12.29 ± 7.03 in the sepsis+antibiotic+MSCs subgroup, as compared with 42.3 ± 8.18 in the sepsis subgroup (*P* = 0.007), 35.77 ± 16.80 in the sepsis+antibiotic subgroup (*P* = 0.016) and 37.80 ± 22.96 in the normal group (*P* = 0.025). ②the protein concentration of bronchoalveolar lavage fluid in the sepsis+antibiotic+MSCs subgroup was less than that in the sepsis subgroup at 24h, (0.41 ± 0.24 vs. 1.17 ± 0.57 mg/ml, *P* = 0.004).

## Conclusions

Intravenous injection of allogeneic MSCs is safe for rats with ARDS induced sepsis. To be administrated in early stage of sepsis, MSCs improve alveolar inflammatory cells infiltration and protein exudation, as well as alveolar congestion and hemorrhage. In addition, there is a potential risk of oxygenation impairment and lung water increase with intravenous injection of MSCs.

## Grant Acknowledgment

Guangdong Natural Science Foundation (S2011040004796)
